# In Memoriam—Dr. Stephen H. Seward

**Published:** 1897-03

**Authors:** 


### IN MEMORIAM—DR. STEPHEN H. SEWARD.

Dr. Stephen H. Seward, the oldest practicing physician in Syracuse, died yesterday (Monday, February 8th), at the family residence, No. 504 West Onondaga Street, in his eighty-seventh year. He leaves one child, a daughter, the wife of G. Lewis Merrell, with whom he has made his home for the last thirteen years. A brother and sister also survive him, the former, Oliver Seward, living at Kankakee, Ill., and the latter, Mrs. C. A. Lewis, of Joliet, Ill.

On his father's side Dr. Seward was related to Hon. William H. Seward, of Auburn, and on his mother's side the relation was to Governor William L. Marcy. Dr. Seward was born September 3d, 1810, at Decatur, Otsego County. His father was one of the contractors who built the Erie Canal. He received a common-school education and then taught school for some five years, and during these years he found time to study medicine and attend the medical department of the then noted Fairfield Seminary, Herkimer County. He graduated and received his diploma in 1837, and commenced practice in Brantford, Canada. From there he removed to Fort Plain, where he married Dollie A. Smith in 1847. Mrs. Seward died at the family residence, on Onondaga Street, 1888.

After one year's practice at Fort Plain Dr. Seward went for one year to Cayuga, Cayuga County, and in about 1845 removed to Liverpool, where he practiced twenty-one years. Like his early distinguished contemporaries, Drs. Lyman Clary, William Hawley, and H. H. Cator, Dr. Seward was an old school physician. Like them, when the new school was winning its first honors, Dr. Seward was induced to test its homœopathic principle. Concerning this test he wrote as follows to a homœopathic publication : "I tested Homœopathy, and after five months I adopted it, and never regretted my choice." In 1868–69 he was made President of the Onondaga County Homœopathic Medical Society.

In 1863 Dr. Seward came to Syracuse and entered into partnership with Dr. H. H. Cator. Later he was a partner of Dr. H. V. Miller, with whom he was associated for five years. For twenty years his office was in the Farmer Block at Warren and Madison Streets.

He was a member of the International Hahnemannian Association and the Central New York Homœopathic Society.

Although eighty-seven years old, Dr. Seward continued in active practice up to Thursday last, February 4th. While visiting patients on that day he caught a severe cold, from the effects of which he did not rally.—*The Syracuse Post, Tuesday, February 9th*, 1897.

